# Preliminary feasibility and efficacy of a brief behavioural treatment for insomnia after acquired brain injury: A case series

**DOI:** 10.1111/jsr.14441

**Published:** 2025-01-09

**Authors:** Maria Gardani, Satu Baylan, Veronika Zouhar

**Affiliations:** ^1^ Department of Clinical and Health Psychology, School of Health in Social Science The University of Edinburgh Edinburgh UK; ^2^ Regional Neuropsychology Service NHS Greather Glasgow and Clyde Glasgow UK; ^3^ NHS Lothian, Royal Edinburgh Hospital Edinburgh UK

**Keywords:** acquired brain injury, brief behavioural intervention, insomnia, sleep

## Abstract

Insomnia after acquired brain injury (ABI) is common and can negatively impact an individual's rehabilitation, recovery, and quality of life. The present study investigated the feasibility and preliminary efficacy of a Brief Behavioural Treatment for Insomnia (BBTI) in a community sample following ABI. Ten participants were recruited. Seven participants attended four weekly sessions of BBTI and kept a daily sleep diary. Participants completed a semi‐structured sleep interview at baseline and self‐report measures of sleep, anxiety, and depression pre‐ and post‐treatment as well as a treatment acceptability questionnaire post‐treatment. Follow‐up data were collected at 1‐, 2‐, and 3‐months post‐treatment. Visual analyses of the data were performed on a case‐by‐case basis. Five of the seven participants (71%) no longer met the criteria for insomnia disorder on the Sleep Condition Indicator (SCI) post‐treatment. Treatment effects on sleep outcomes were either maintained or augmented at follow‐ups. BBTI was found to be well tolerated, as evidenced by the high overall retention rates (70%) and positive feedback on the treatment acceptability questionnaire. These results provide preliminary evidence of BBTI being both feasible to use and potentially efficacious in individuals with post‐brain‐injury insomnia. Larger‐scale randomised controlled trials are needed to establish the effectiveness of BBTI following ABI.

## 1 INTRODUCTION

Acquired brain injury (ABI) is one of the leading causes of neuro‐disability worldwide, leaving individuals with profound physical, cognitive, and mental consequences post‐injury (Hilkens et al., 2024). Sleep disturbances, including insomnia disorder, are among the most frequent complaints for people following traumatic brain injury (TBI) (Mathias & Alvaro, 2012). Insomnia, one of the most common and debilitating sleep disorders, is characterised by difficulty falling asleep, staying asleep, and/or waking up too early despite ample opportunity to sleep (American Psychiatric Association, 2013). The prevalence of insomnia disorder and symptoms is higher in stroke survivors (Baylan et al., 2020) and those with a mild TBI (Montgomery et al., 2022) than in the general population (Ohayon, 2002).

A number of possible mechanisms may contribute to the high incidence and prevalence of sleep disturbances and insomnia following brain injuries, such as disruptions to the sleep‐regulating brain regions and/or the associated pathways and neurotransmitter systems (Ponsford et al., 2012). In hospitalised patients, environmental factors such as room sharing, noise, and light may also contribute to the onset and maintenance of insomnia and insomnia symptoms at the acute phase post‐injury (Gellerstedt et al., 2014) or during rehabilitation (Amato & Anthony, 2020). Psychological factors such as worry about sleep and diminished perception of control may also play a role in insomnia following ABI (Verkaik et al., 2024). A bidirectional relationship is often reported between insomnia and mental health disorders with poor sleep quality being more common and independently associated with post‐stroke anxiety (Xiao et al., 2020). The consequences of untreated sleep disruption are well evidenced in healthy individuals, with numerous studies demonstrating the impact of poor sleep on cognition, mood, learning, memory, and brain plasticity (Walker & Stickgold, 2006) that constitute the core modalities of neuropsychological rehabilitation and recovery after a brain injury. Emerging evidence demonstrates that the presence of a sleep disorder post‐ABI is associated with negative recovery outcomes, including depression, disability, and fatigue (Fulk et al., 2020; Tang et al., 2015) in addition to low functional and motor outcomes at admission and discharge from rehabilitation (Fleming et al., 2020). In animal models of ischaemic stroke, such neurological impairments were improved by experimental manipulation of sleep states (Facchin et al., 2020), suggesting that sleep may be a primary driver of functional recovery post brain injury. Despite these mechanistic links of sleep to ABI recovery and the well‐established associations of insomnia with outcome in human stroke survivors, sleep is one of the least explored mechanisms in stroke functional recovery (Duss et al., 2017).

Nonpharmacological interventions for sleep, such as Cognitive Behavioural Therapy for Insomnia (CBT‐I), typically delivered over 6–8 sessions, show promising preliminary results for insomnia in stroke survivors (Ford et al., 2023; Herron et al., 2018). However, the length of treatment and the lack of access to psychological therapy pose significant limitations to these approaches (Koffel et al., 2018). Furthermore, participants had difficulty with adhering to the CBT‐I protocol due to physical and cognitive complications following stroke (Ford et al., 2023), a finding that was supported by a qualitative investigation (Smejka et al., 2022) indicating that behavioural interventions may be more suitable for this population. Alternative and shorter behavioural interventions for insomnia have been developed (for review see McLaren et al., 2023) such as Brief Behavioural Treatment for Insomnia (BBTI) (Troxel et al., 2012). BBTI is based primarily on the principles of sleep restriction and stimulus control, which constitute the behavioural components of CBT‐I. Previous evidence shows that stimulus control and sleep restriction (as part of a CBT‐I intervention) resulted in rapid benefits for community patients post‐TBI in a case series study (Ouellet & Morin, 2007).

The feasibility and efficacy of BBTI have been successfully demonstrated in diverse populations. For instance, Buysse et al. (Buysse et al., 2011) explored the efficacy of BBTI in a sample of older adults with chronic insomnia. The experimental group received a 4‐week‐long BBTI delivered by a nurse with no experience in sleep medicine, and the control group was provided with educational material for self‐study. At post‐treatment, compared with the control group the BBTI condition group showed improved outcomes on self‐reported sleep questionnaires, sleep diaries, and actigraphy and met insomnia disorder criteria less often. These improvements were well maintained at a 6‐month follow‐up. A promising response to BBTI has also been found in individuals living with HIV (Buchanan et al., 2018), in combat‐exposed military personnel (Germain et al., 2014), and in clients with treatment‐resistant insomnia (Wang et al., 2016).

Following the same 4‐week BBTI protocol the main objective of the present study was to explore the feasibility and preliminary efficacy of the brief behavioural intervention for insomnia in a case series sample with insomnia after brain injury including people with stroke and traumatic brain injury (TBI).

## 2 METHODS

### 2.1 Design and ethics

A case series design was adopted. The University of Glasgow College of Science and Engineering Ethics Committee reviewed and approved the study (Ref: 300200056).

### 2.2 Participants

#### 2.2.1 Recruitment, inclusion, and exclusion criteria

Participants were recruited through a local branch of a UK‐wide brain‐injury charity (Headway) from 10 November 2016 to 10 November 2017. No monetary reward for participation was offered. Inclusion criteria were as follows: (a) over 18 years old; (b) have sustained a brain injury as an adult (>18 years old); (c) presence of insomnia based on screening with the Sleep History Interview. Participants taking hypnotic medication were also eligible, provided that they had not changed their medication in the 3 weeks prior to recruitment. Exclusion criteria were: (a) level of English did not allow them to follow instructions; (b) significant cognitive difficulties – based on their charity support workers – that would have prevented engagement with the study protocol; (c) presence of another self‐reported sleep disorder, such as sleep apnea; (d) presence of self‐reported severe mental health condition, such as bipolar disorder; (e) receiving a psychological intervention for their sleep problem; (f) presence of a self‐reported neurological disorder, such as epilepsy or a neurodegenerative condition.

### 2.3 BBTI intervention

Brief Behavioural Treatment for Insomnia (BBTI) is a four‐session behavioural insomnia intervention, with two sessions typically delivered in person and two over the phone (Troxel et al., 2012). BBTI is based on the well‐established behavioural principles of sleep restriction and stimulus control (Perlis et al., 2005) but also includes education on sleep regulation and hygiene (Buysse et al., 2011). These components provide a rationale for the four main rules of BBTI, specifically: (1) Reduce time in bed, (2) Don't go to bed unless sleepy, (3) Don't stay in bed unless asleep, and (4) Wake up at the same time every day, no matter how much you slept the night before (Troxel et al., 2012).

**FIGURE 1:**
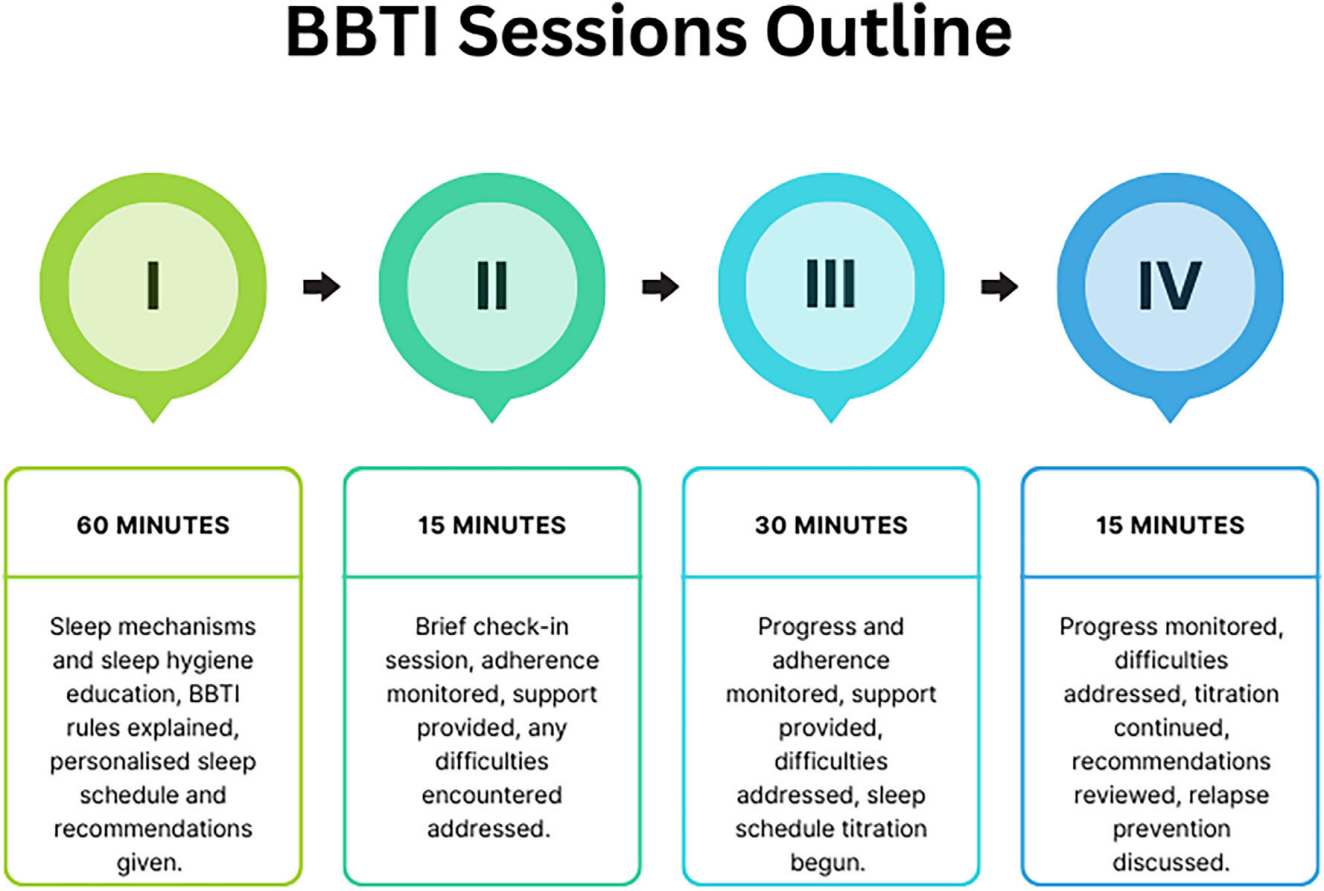
BBTI sessions outline.

The intervention was tailored to the needs of the participants with respect to their post‐brain injury memory and cognitive abilities and thus deviated from the original planned procedure as outline by Troxel and colleagues (Troxel et al., 2012). More specifically, all sessions were delivered in person in a convenient place and time for participants, compared with having sessions II and IV over the phone. The location was the charity premises and the time was during their usual visiting times as it was the most convenient for all participants to attend. Secondly, session I was delivered to small groups (2–3 participants) rather than individually due to research resources. Third, discussion on how and why sleep is affected following a brain injury was included in the educational component of session I. Participants were provided with a paper workbook and were asked to keep a daily sleep diary throughout the intervention and to address frequent post‐brain‐injury memory problems, participants received daily morning reminders to complete their sleep diary. The content of the individual sessions is outlined in a schematic graph in Figure 1.

### 2.4 Questionnaire measures

#### 2.4.1 Sleep

A semi‐structured Sleep History Interview, was developed by the research team to capture rich in‐depth information on the participants' sleep routine, identify the presence of sleep disorders and any consequences of sleep disturbances on daily functioning (Gardani et al., 2015). This interview established the subjective impact of the brain injury on the individual's sleep.

A sleep diary (Carney et al., 2012) was used to collect daily information on participants' sleep patterns (10 items) and quality (2 items). To rate the subjective sleep quality, a scale from 0 to 4 was used, with higher scores indicating better sleep quality. Sleep diary parameters were calculated by the researchers throughout the intervention to inform recommendations given. These were defined as: TWT‐ total wake time, i.e. sleep onset latency, and any wakefulness after sleep onset; SE − sleep efficiency, i.e. total sleep time over time in bed, multiplied by 100 and SQ‐ subjective sleep quality.

Sleep quality was assessed using the Pittsburgh Sleep Quality Index (PSQI); (Buysse et al., 1989), a well‐established self‐report measure validated for use with people after brain injury (Fichtenberg et al., 2001). Scores on each of the seven clinically relevant sleep quality components range from 0 to 3, yielding a global score with range from 0 to 21, where higher scores indicate poorer sleep quality. In the brain injury population, a cut‐off score of 8 has been recommended to discriminate good and poor sleepers (Kaufmann et al., 2019).

The Sleep Condition Indicator (SCI); (Espie et al., 2014) was employed to obtain an assessment of insomnia symptoms. The SCI comprises 8 items with scores for each item ranging from 0 to 4 with higher total scores indicating better sleep. A score of 16 or higher is indicative of no insomnia disorder.

#### 2.4.2 Depression and anxiety

The Hospital Anxiety and Depression Scale (HADS); (Zigmond & Snaith, 1983) was used to monitor changes in anxiety and depression over the course of the study. The HADS is composed of seven depression and seven anxiety items, each rated on a scale from 0 to 3. The level of depression and anxiety is classed as normal (total score of 0–7), mild (total scores of 8–10), moderate (total scores of 11–14), and severe (total scores of 15–21) and is validated as a self‐report measure for people with TBI (Dahm et al., 2013).

#### 2.4.3 Treatment acceptability

A Treatment Acceptability and Credibility Questionnaire was developed by the authors for the purposes of this study in order to gain a better understanding of the participants' overall experience with the intervention. The questionnaire covered the pertinent elements of the intervention, including the perceived therapists' level of knowledge and style, acceptability of the mode and location of delivery, length of the intervention, ease of following materials, the main treatment principles and specific recommendations, and their perceived effectiveness of the intervention. Participants were asked to rate 13 closed‐ended questions on a scale from 0 to 7, with higher scores indicating greater satisfaction with the intervention. Further, three open‐ended questions asked about liked and disliked aspects of the intervention and additional comments.

### 2.5 Procedure

#### 2.5.1 Baseline

Interested participants were screened for eligibility and those meeting inclusion criteria received a detailed participant information sheet about the study before written consent was obtained. Pre‐treatment measures were collected by VZ and MG, including demographic characteristics, semi‐structured Sleep Interview, PSQI, SCI, and HADS. Participants were provided with a paper copy of the sleep diary. They were asked to complete it daily in the morning and bring it to the first intervention session in a week's time. As daily completion was essential, participants were offered daily text message reminders.

#### 2.5.2 Intervention sessions (I‐IV)

The intervention was delivered over 4 weeks (one session per week) after baseline in accordance with the protocol. The intervention was delivered by the first author (MG) who had training and extensive research experience delivering CBT‐I and subsequently by the third author (VZ) who was also trained.

#### 2.5.3 Post‐treatment and follow‐up sessions

A week after finishing the intervention, participants completed the post‐treatment measures, consisting of the same questionnaire battery as pre‐treatment. Additionally, participants filled in the Treatment Acceptability and Credibility Questionnaire. Follow‐ups were scheduled for 1 month, 2 months and 3 months to monitor progress. At these sessions, the same battery of measures was completed and sleep diary data for 7 days were obtained at the end of each follow‐up session.

### 2.6 Treatment response

The criteria of response to treatment were based on previously published case series utilising BBTI (Germain et al., 2006). Clinical judgements of treatment response were made if participants were showing an improvement on at least two of the following criteria: (1) PSQI score of <8, (2) SCI score of ≥16, (3) TWT ≤30 min, SE ≥85%, SQ ≥2 (any two out of three), (4) no current self‐reported sleep problems. In this case they were characterised as responders. Further, for a moderate response to the intervention at least two of the following criteria needed to be met: at least a 3‐point improvement on PSQI, at least a 5‐point improvement on SCI, 10% improvement in two out of three sleep diary parameters (TWT, SE, SQ) compared with pre‐treatment. Those that did not reach the above stated criteria were classified as non‐responders.

## 3 RESULTS

### 3.1 BBTI feasibility

#### 3.1.1 Retention and attendance

Ten individuals approached the researchers, and all were found to be eligible to participate. One participant withdrew before starting the intervention, and two withdrew after the first BBTI session (I), resulting in seven (*n* = 7) participants completing the study with an overall study drop‐out rate of 30% and intervention dropout rate of 22.22%. One participant paused treatment due to a worsening of a family member's medical condition and resumed when their condition stabilised. All participants completed all BBTI sessions.

##### Participant characteristics

The sample consisted of six males and one female, aged 41–70 years (median = 54 years). All participants were white; one was living with a partner and children, two with children only and four were living alone. Participants' qualifications ranged from upper secondary to postgraduate. One participant was returning to work at the time of the study whilst the rest had no plans for return to work. Four participants sustained a TBI (two due to accident, one due to assault, one due to fall) and three an ABI (one due to brain tumour, one due to ischaemic stroke, and one due to anoxia). Time since injury was 2–32 years (median = 4 years). All participants reported being hospitalised following their brain injury and five participants received post‐brain injury rehabilitation (Table 1). All participants reported experiencing at least five common consequences of brain injury other than sleep problems (e.g. sensory, memory, mood disturbances). Comorbid physical conditions were present in three participants, two disclosed previously treated mental health disorders. All were taking at least one, but some as many as six different types of prescribed medication.

**TABLE 1:** Participant demographic and injury characteristics.

	Age	Sex	Type of BI	Time since BI (years)	Hospitalisation after BI	Rehabilitation after BI
P1	49	M	TBI	3	Y	Y
P2	70	M	ABI	4	Y	N
P3	57	M	TBI	32	Y	Y
P5	41	F	TBI	2	Y	N
P7	54	M	ABI	3	Y	Y
P8	42	M	ABI	5	Y	Y
P9	55	M	TBI	10.5	Y	Y

Abbreviations: ABI, acquired brain injury; BI, brain injury; F, female; M, male; N, no; P, participant; TBI, traumatic brain injury; Y, yes, N, no.

### 3.2 Sleep diary completion

Compliance with daily sleep diary completion was high. A mean number of 48.71 (SD = 12.54) sleep diary entries were extracted per participant (range = 28–63 days). This range was a result of additional sleep diaries being available for those who had completed follow‐ups and for those who had treatment spread over a longer period due to pausing or rescheduling a session.

### 3.3 Treatment acceptability and credibility

Item‐by‐item mean scores for the Likert‐scale questions on the Treatment Acceptability and Credibility Questionnaire ranged from 6.29 to 6.71 out of 7, indicating high subjective satisfaction with the treatment format, procedures, content, and effectiveness. The answers provided to the three subsequent open‐ended questions were also indicative of high satisfaction with the delivered intervention. Specifically, only one participant identified a treatment component they particularly struggled with (P9 − no naps) and all participants gave informative examples of treatment components they liked (e.g. clear sleep schedules − P2, P8).

### 3.4 BBTI efficacy

#### 3.4.1 Sleep outcomes

##### Sleep diaries

Mean total wake time (TWT), sleep efficiency (SE), and sleep quality (SQ) were recorded for all participants throughout the duration of the study. Visual analyses of changes in mean sleep parameters were performed on a case‐by‐case basis and are shown in Table 2.

**TABLE 2:** Sleep diary parameters at pre‐ and post‐treatment and at 1‐, 2‐, and 3‐month follow‐ups.

	TWT					SE					SQ				
	Pre	Post	FU1	FU2	FU3	Pre	Post	FU1	FU2	FU3	Pre	Post	FU1	FU2	FU3
TBI															
P1	35.36	19.29	19.29	15.71	38.57	74.32	81.85	84.00	81.76	79.10	2.00	2.00	2.00	2.00	2.00
P3	100.72	28.58	34.29	30.00	–	65.88	86.50	84.26	90.83	–	1.64	1.93	1.71	1.79	–
P5	49.28	51.42	–	–	–	85.16	83.16	–	–	–	1.00	0.83	–	–	–
P9	10.00	10.00	10.00	10.00	–	97.15	97.06	97.32	97.37	–	0.93	1.00	1.57	2.00	–
ABI															
P2	180.00	34.28	25.00	26.66	32.14	35.63	82.18	85.73	75.51	87.72	1.39	2.14	2.18	2.14	2.14
P7	170.72	109.29	10.00	4.29	–	92.49	74.36	96.23	95.43	–	0.14	1.36	2.50	2.71	–
P8	73.34	50.36	–	–	–	65.87	86.47	–	–	–	1.79	2.93	–	–	–

*Note*: TWT in minutes, SE in %, SQ scored on a 0–4 scale, with higher scores indicating better sleep quality.

Abbreviations: Pre, pre‐treatment; Post, post‐treatment; SE, sleep efficiency; SQ, sleep quality; TWT, total wake time; FU1/2/3, 1/2/3‐month follow‐up.

#### 3.4.2 Sleep questionnaires

A substantial pre‐ to post‐treatment change in self‐reported sleep measures was present following BBTI for most participants, with the exception of P5 (Table 4). PSQI scores showed a general decrease, with six of the seven participants scoring 8 or less post‐treatment, indicative of good sleep quality. This was generally well‐maintained at follow‐up. Further, SCI scores showed an increase across all participants, indicating improvement in insomnia symptoms, with five of the seven participants no longer meeting the threshold for an insomnia disorder post‐treatment. These gains were either maintained or improved at follow‐up.

#### 3.4.3 Mood

Co‐occurring changes in HADS‐A scores were present, with one participant showing a reduction in anxiety symptoms from mild to normal level, one from moderate to normal level, one from moderate to mild level, and one from severe to moderate level post‐treatment. There were also modest improvements in HADS‐D scores for some participants, with one participant showing a reduction from moderate to normal level, two from moderate to mild level, and one from mild to normal level of depression. Improvements in subjective mood measures were mostly well maintained, and a few participants showed further reductions in anxiety and depression scores, particularly at 1‐ and 2‐month follow‐ups (Table 3).

**TABLE 3:** PSQI, SCI, HADS scores at pre‐ and post‐treatment and at 1‐, 2‐, and 3‐month follow‐ups.

	Sleep										Mood									
	PSQI					SCI					HADS‐A					HADS‐D				
	Pre	Post	FU1	FU2	FU3	Pre	Post	FU1	FU2	FU3	Pre	Post	FU1	FU2	FU3	Pre	Post	FU1	FU2	FU3
TBI																				
P1	10	6	6	5	8	20	31	31	31	30	2	2	2	2	1	10	5	8	8	10
P3	9	4	6	3	–	9	28	24	30	–	2	1	6	1	–	6	1	6	0	–
P5	8	8	–	–	–	12	13	–	–	–	13	7	–	–	–	11	9	–	–	–
P9	11	6	4	4	–	6	18	28	29	–	9	6	5	1	–	10	10	8	7	–
ABI																				
P2	14	5	3	5	4	6	27	30	27	29	12	10	5	7	10	10	9	6	7	8
P7	10	9	3	3	–	4	13	32	29	–	6	3	4	3	–	11	9	4	4	–
P8	14	6	–	–	–	4	25	–	–	–	16	12	–	–	–	11	7	–	–	–

Abbreviations: FU1/2/3, 1/2/3‐month follow‐up; HADS‐A/D, Hospital Anxiety and Depression Scale, Anxiety/Depression subscale; Post, post‐treatment; Pre, pre‐treatment; PSQI, Pittsburgh sleep quality index; SCI, sleep condition indicator; SE, sleep efficiency; SQ, sleep quality; TWT, total wake time.

#### 3.4.4 Treatment response

Using the predetermined criteria, five out of seven participants were responders (P1, P2, P3, P8, P9) demonstrating a significant improvement post‐treatment. This change was maintained by P1, P2, P3, and P9 at all available follow‐ups (Table 4). For P8 no follow‐up data were available.

**TABLE 4:** Clinical significance of outcomes at post‐treatment and at 1‐, 2‐, and 3‐month follow‐ups.

	PSQI score <8				SCI score ≥ 16				TWT ≤30 min, SE ≥85%, SQ ≥2^a^				Current sleep problems^b^				Clinically significant improvement post‐treatment
	Post	FU1	FU2	FU3	Post	FU1	FU2	FU3	Post	FU1	FU2	FU3	Post	FU1	FU2	FU3	
TBI																	
P1	YES	YES	YES	NO	YES	YES	YES	YES	YES	YES	YES	NO	YES	YES	YES	YES	**YES**
P3	YES	YES	YES	–	YES	YES	YES	–	YES	NO	YES	–	Unsure	Unsure	YES	–	**YES**
P5	NO	–	–	–	NO	–	–	–	NO	–	–	–	NO	–	–	–	**NO**
P9	YES	YES	YES	–	YES	YES	YES	–	YES	YES	YES	–	NO	NO	YES	–	**YES**
ABI																	
P2	YES	YES	YES	YES	YES	YES	YES	YES	NO	YES	YES	YES	NO	YES	YES	YES	**YES**
P7	NO	YES	YES	–	NO	YES	YES	–	NO	YES	YES	–	NO	YES	YES	–	**NO**
P8	YES	‐	–	–	YES	–	–	–	YES	–	–	–	NO	–	–	–	**YES**

^a^ At least two out of three need to be met.

^b^ A “no” answer to the question “Do you currently have problems with your sleep?” in the Semi‐structured Sleep Interview.

P7 met the criteria for moderate response post‐treatment and reached the criteria for responder at all available follow‐ups.

#### 3.4.5 Case study

In order to give a comprehensive illustration of the effects of the intervention, we included the following case study. Participant 2 (P2) was a 70‐year‐old male, retired and living alone, with upper secondary qualification. He had no diagnosed physical or mental illnesses, but was regularly taking simvastatin, amlodipine, and occasionally co‐codamol. He sustained a moderate–severe ABI due to a tumour that was surgically removed 4 years prior to his participation in the study. He had spent 5 weeks in hospital but was not offered rehabilitation post‐surgery.

P2's primary complaint was difficulty falling asleep, which affected him seven nights a week. He experienced some night awakenings and did not feel refreshed after a typical night. He perceived his poor sleep to significantly impact his daytime functioning. He disclosed sometimes taking naps and using a laptop in bed. The individualised treatment recommendations included reducing time in bed from nearly 13 h per night to seven, as well as eliminating daytime napping and the use of their laptop in bed.

His PSQI scores decreased from 14 to 5, indicative of good sleep quality post‐treatment. Further, his SCI score increased from 6 to 27, indicating no presence of insomnia disorders after treatment. Co‐occurring changes in HADS‐A scores (12–10) were present, reflecting a non‐clinically significant reduction in anxiety symptoms with a change in symptom severity classification from moderate to mild. The HADS‐D score changed minimally and remained mild post‐treatment. Improvements in subjective sleep measures were maintained, while anxiety and depression scores showed further reductions, particularly at 1‐ and 2‐month follow‐ups.

**FIGURE 2:**
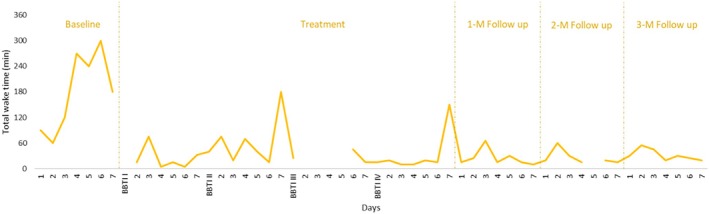
Changes in total wake time over the course of the study (P2).

**FIGURE 3:**
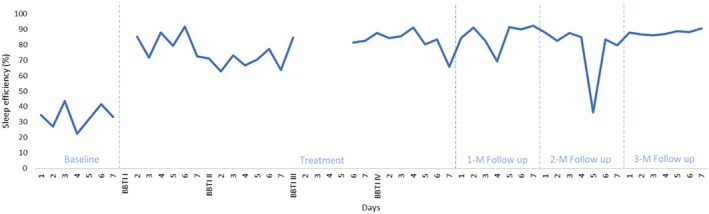
Changes in sleep efficiency over the course of the study (P2).

**FIGURE 4:**
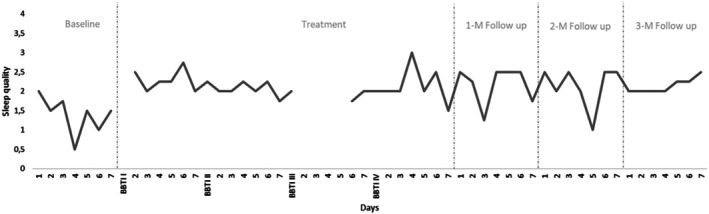
Changes in sleep quality over the course of the study (P2).

Using the criteria for visual analysis of time series plots suggested by Kazdin (Kazdin, 2019), it was noted that for all three parameters, change was discontinuous rather than gradual, with a clearly perceptible decreasing trend in TWT, and increasing trends in SE and SQ. Overall, improvements remained well‐maintained at all 1‐, 2‐, and 3‐month follow‐ups, despite the one very poor night of sleep visible at 2‐month follow‐up, likely due to alcohol consumption. Further, a clear reduction in inter‐night variability was observed for all three parameters towards the end of the study period. Changes in sleep diary parameters throughout the study are depicted in Figures 2, 3, 4 respectively.

## 4 DISCUSSION

This is the first study, with a case series design, to demonstrate the feasibility and potential efficacy of Brief Behavioural Treatment for Insomnia for individuals after brain injury on sleep outcomes for all participants, including sleep quality with change evident for the majority of participants on questionnaire measures with accompanying improvement in mood.

More specifically, regarding the feasibility of BBTI in this sample, most participants tolerated the intervention well. The drop‐out rate from the start of intervention to post‐intervention was 22.22%, which is comparable to other intervention studies conducted in this population (Zollman et al., 2012) as well as BBTI investigations in other groups (McLaren et al., 2023). Further, seven of the nine participants who commenced the intervention attended all sessions and completed all sleep diaries. Moreover, the feedback given in the Treatment Acceptability and Credibility Questionnaire pointed to very high satisfaction with all treatment components, exceeding the already positive ratings BBTI received in other populations (Buchanan et al., 2018).

As far as the preliminary efficacy of BBTI in this sample is concerned, five out of seven participants demonstrated clinically significant sleep improvement post‐treatment, one participant showed moderate response and one participant showed nonresponse, according to the predetermined treatment response criteria. While no universal standard for classifying insomnia treatment response exists (Riemann et al., 2023), other BBTI studies used criteria similar to ours and reported relatively comparable ratios (Germain et al., 2006). The proportion of participants with brain injury and insomnia demonstrating a clinically significant response to CBT‐I reported in Ouellet and Morin (Ouellet & Morin, 2007) (73%) was comparable to our results. This indicates that for people with brain injury a shorter and more easily disseminated intervention, such as the BBTI, may be a good alternative to the better‐established CBT‐I in terms of efficacy.

Participants in the treatment response category presented markedly improved changes in both sleep and insomnia questionnaire measures (PSQI and SCI) as well as sleep diary parameters (TWT, SE, SQ). Moreover, the inter‐night variability in these parameters, which is typical of insomnia (Suh et al., 2012) was reduced in most cases. Sleep diary parameter changes were mostly gradual (except for P2) and appeared within 1 or 2 weeks after starting the intervention. As this is the same time frame for improvement noted in Ouellet and Morin (Ouellet & Morin, 2007) it is possible that observable positive effects by the second week of treatment may serve as a reliable predictor of the ultimate treatment response. Furthermore, where follow‐up data were available, sleep‐related improvements were well‐maintained, or indeed further augmented, which provides evidence for the durable effects of BBTI already established by previous research (Buysse et al., 2011), however, the sample size is too small, in the context of case series methodology, for firm conclusions to be drawn.

Sleep‐related improvements were in most cases accompanied by a decline in self‐reported anxiety and depression symptoms, mirroring the findings of Fuller et al. (Fuller et al., 2016). The general association of insomnia with mental health complaints is well‐documented, yet its directionality remains unclear (Fernandez‐Mendoza & Vgontzas, 2013). Consequently, it is difficult to ascertain whether in the present study sleep improvements led to anxiety and depression reductions or reductions in anxiety and depression brought about improvements in sleep or whether each operated relatively independently. This latter option tentatively emerges as most likely, considering the two participants who did not show clinically significant sleep improvements (P5, P7) both presented anxiety and depression symptom reductions.

While these findings are promising overall, the characteristics of the selected design (particularly the lack of a control group) do not permit establishing with certainty that improvements arose due to the administered insomnia treatment. Several alternative explanations must be considered (Kazdin, 2024). First, if we assumed sleep disturbances spontaneously improved as brain recovery progressed, then the passing of time since injury could have led to the alleviation of reported sleep complaints. Yet, based on the participants' self‐report, spontaneous improvements of sleep disturbances since the injury ranged from non‐existent to minimal and the insomnia symptoms had been present for a median of 4 years. It is thus implausible that the observed marked change occurring over the course of a few weeks could be solely due to the passing of time. Future studies should consider including a control group or implement single case experimental design methodology to increase confidence of any observed changes being due to the effects of the intervention.

Second, while face‐to‐face client‐therapist contact was limited to once per week, participants received daily text reminders which contained some personalised elements. Thus, the virtually daily study contact could have induced the symptom improvements, rather than the BBTI itself. However, self‐reported adherence to particular treatment recommendations typically preceded observed effects. Further, participant 5 who complained of insomnia despite already practising most of the BBTI recommendations and emphasised liking the regular contact did not show improvement on sleep parameters merely through contact with the therapist. Therefore, despite constituting an important active element to any talking therapy, it is unlikely that contact via text messaging could solely account for the observed treatment effects.

Two participants did not show clinically significant treatment improvements. For P7 (demonstrating moderate response), modest positive effects appeared towards the end of the treatment course. P7 presented with further improvements in the weeks following the end of the treatment, reaching the criteria for a responder at 1‐month and 2‐month follow‐ups. Notably, for P7 the timing of the treatment coincided with several adverse life events, which may have masked the benefits of the intervention at that time, with these benefits fully appearing when some time since the adverse events has passed.

Only P5 showed nonresponse to treatment, albeit presenting a high motivation to change and being one of the only participants with significant family support. P5 is also the only woman in our study, however, previous evidence has demonstrated the efficacy of the intervention in female participants (Buysse et al., 2011). In this case, it is plausible that BBTI failed to produce sleep improvements as the participant was already instinctively practising most of the core treatment elements. Their total sleep time was already short and any further restriction would have violated the recommended safe minimum. As discussed by Buchanan et al. (Buchanan et al., 2018) and in accordance with stepped‐care model of insomnia (Baglioni et al., 2023), there may be a small subset of clients for whom short and purely behavioural therapy is not sufficient and such clients should be considered for the longer CBT‐I delivered by a sleep specialist.

### 4.1 Limitations

Several limitations to the present study must be considered, such as lack of access to medical records. All information regarding brain injury characteristics was obtained through self‐report, which could have contained important distortions, especially considering any post‐brain‐injury memory problems (Maeshima & Osawa, 2021). Individuals with brain injury have been shown to overestimate their sleep difficulties when subjective reports were compared with objective data (Gardani et al., 2015; Ouellet & Morin, 2006). The current sample consisted of participants attending Headway support services hence it is possible that their insomnia symptoms did not impact their social engagement. Additionally, participants' responses to HADS demonstrate low levels of depression. As depression is highly comorbid with insomnia (Riemann et al., 2020) and has a high prevalence in those following a brain injury (Garrelfs et al., 2015) future studies should explore whether participants with moderate to severe symptoms of depression may benefit from the intervention.

Future studies should include objective measures of sleep and circadian patterns, such as actigraphy or ambient recorded polysomnography and utilise published Reliable Change Index scores to assess clinically significant change post‐treatment. Lastly, longer follow‐ups may have been more informative as to the long‐term maintenance of the benefits achieved. Follow up data were not collected for a few participants due to difficulty to maintain contact.

### 4.2 Future directions, implications and conclusions

The promising findings of our study expand on the existing evidence base suggesting that brief, non‐specialist‐delivered and purely behavioural therapies can yield positive results in people with insomnia after brain injury. As the study did not include participants with a specific brain injury but rather people with stroke, TBI, and tumour further research should investigate the use of this treatment for insomnia in each population group employing single case experimental design and randomised controlled trial protocols.

## AUTHOR CONTRIBUTIONS

**Maria Gardani:** Conceptualization; methodology; supervision; writing – original draft; writing – review and editing. **Satu Baylan:** Writing – review and editing. **Veronika Zouhar:** Methodology; data curation; visualization; writing – original draft; writing – review and editing.

## Data Availability

The data that support the findings of this study are available on request from the corresponding author. The data are not publicly available due to privacy or ethical restrictions.
